# Smartphone addiction and learning engagement: a parallel mediation model of time management disposition and academic burnout among nursing undergraduates

**DOI:** 10.3389/fpsyg.2026.1927919

**Published:** 2026-09-08

**Authors:** Jingui Huang, Yumei Shi, Feifei Li, Pei He, Junkai Liu, Haimei Zhang

**Affiliations:** 1 Department of Medical Oncology, Chongqing University Cancer Hospital, Chongqing, China; 2 Department of Obstetrics, The First Affiliated Hospital of Chongqing Medical University, Chongqing, China; 3 Department of Nursing, People’s Hospital of Ningxia Hui Autonomous Region, Yinchuan, China; 4 Organization Department, Huanggang Polytechnic University, Huanggang, Hubei, China; 5 Department of Nursing, Chongqing University Cancer Hospital, Chongqing, China

**Keywords:** academic burnout, learning engagement, nursing undergraduates, smartphone addiction, time management disposition

## Abstract

**Background:**

We aimed to explore the relationship between smartphone addiction and learning engagement among nursing undergraduates, and to analyze the parallel mediating effects of time management disposition and academic burnout in this relationship.

**Methods:**

In this multi-center cross-sectional study, we used a convenience sampling method to recruit nursing undergraduates from five universities in Chongqing Municipality, Hubei province, and the Ningxia Hui Autonomous Region. Data were collected using a general information questionnaire, the Smartphone Addiction Scale, Time Management Disposition Scale, Academic Burnout Scale, and Learning Engagement Scale. Descriptive statistics and correlation analyses were performed using IBM SPSS version 25.0, and structural equation modeling was used to test the mediating effects.

**Results:**

Smartphone addiction was significantly negatively correlated with learning engagement (*r* = −0.551). Time management disposition was significantly positively correlated with learning engagement (*r* = 0.692) and significantly negatively correlated with smartphone addiction (*r* = −0.456). Academic burnout was significantly negatively correlated with learning engagement (*r* = −0.603). Structural equation modeling revealed that time management disposition (β = −0.274, 95% *CI*: −0.203 to −0.118) and academic burnout (β = −0.071, 95% *CI*: −0.078 to −0.006) had partial mediating effects on the interaction between smartphone addiction and learning engagement. The two pathways constituted a parallel mediating effect, accounting for 61.17% of the total effect.

**Conclusion:**

Smartphone addiction was significantly associated with learning engagement, and time management disposition and academic burnout acted as significant partial mediators in this association. Comprehensive strategies including time management training, academic burnout interventions, and smartphone use guidance in universities may help mitigate the negative impacts of smartphone addiction and improve nursing undergraduates’ learning engagement.

## Introduction

In view of rising healthcare demands and a global shortage of nurses, undergraduate nursing education, which is the primary foundation for training high-quality nursing professionals, directly influences the quality of future clinical care (Fu et al., 2022). Learning engagement is a key indicator of educational quality and academic outcomes (Newmann, 1992), that captures the time and effort students devote to academic and practical activities, reflecting their recognition of learning value and immersive involvement in the learning process (Schaufeli et al., 2002b; Wang et al., 2024). It is a multidimensional construct encompassing cognitive, emotional, and behavioral engagement (Schaufeli et al., 2002b), serving as a core driver linking knowledge acquisition to competence development (Guo et al., 2025; Sun et al., 2023; Xing, 2026). For nursing undergraduates in particular, learning engagement is not only a reflection of their subjective initiative but also a vital factor for enhancing learning abilities, advancing professional growth, and ultimately improving the quality of patient care. However, nursing students face multiple academic, clinical, and interpersonal stressors (Lavoie-Tremblay et al., 2022), making their learning engagement levels suboptimal (Liu et al., 2025, 2026). Therefore, exploring the influencing factors and internal mechanisms of learning engagement among nursing undergraduates is essential for optimizing nursing education models and supporting students’ long-term academic development.

Smartphone addiction, defined as compulsive, uncontrollable use despite adverse consequences (Sunday et al., 2021), has emerged as a novel risk factor associated with student learning engagement. It is classified as a non-chemical behavioral addiction, and is a common concern among nursing students (Alreshidi, 2026). The prevalence of smartphone addiction among nursing students ranges from 40% to 50% (Zhou et al., 2024). It impairs sleep quality and attention span (Uzunçakmak et al., 2022; Zhou et al., 2024) and exacerbates psychological stress and emotional problems (Nikolic et al., 2023), thereby hindering active participation in learning. Previous studies have reported a significant negative correlation between smartphone addiction and learning engagement; however, the underlying mechanisms in nursing undergraduates remain insufficiently explored.

Time management disposition is an important component that links smartphone addiction to learning engagement. According to the theory of time structure (Claessens et al., 2007), organized and meaningful time allocation supports the maintenance of healthy and positive behaviors, while the lack of temporal structure leads to procrastination and disorganization. Excessive smartphone use disrupts individuals’ time perception and daily routines (Yuan et al., 2023), weakening their ability to plan and monitor study time. Given that nursing curricula greatly rely on consistent time management, reduced time management capacity directly impedes the execution of study plans and the maintenance of learning engagement (Liu et al., 2014).

Another key pathway in this relationship may be academic burnout. According to the Conservation of Resources theory (Bon and Shire, 2022), persistent stressors deplete psychological resources, leading to maladaptive outcomes. As a behavioral addiction, smartphone addiction continuously consumes limited cognitive and emotional resources, significantly increasing the risk of academic burnout (Li et al., 2026; Liu, 2023). In turn, academic burnout directly undermines learning engagement by suppressing students’ learning motivation, diminishing their vitality and concentration during learning activities, and reducing their overall enthusiasm for active participation in academic task (Jeon et al., 2022; Zhang et al., 2023).

Despite the valuable achievements of prior studies, several gaps remain to be addressed. First, although previous research has established associations among these variables, for instance, between time management disposition and learning engagement, and between smartphone addiction and academic burnout-these relationships have largely been examined in isolation. Few studies have integrated these variables into a single framework to simultaneously explore the mechanism through which smartphone addiction is associated with learning engagement, leaving the mediating mechanisms and their effects largely unclear. Second, existing evidence has predominantly focused on junior high school and general college students (Liu et al., 2022; Zhang S. et al., 2026; Zhang et al., 2023), with limited attention paid to nursing undergraduates. Nursing students face unique academic and clinical demands that may limit the generalization of findings from other student populations. Third, much of the available evidence is derived from single-region or single-center samples, which may constrain the generalizability of findings to diverse educational and cultural settings.

**FIGURE 1:**
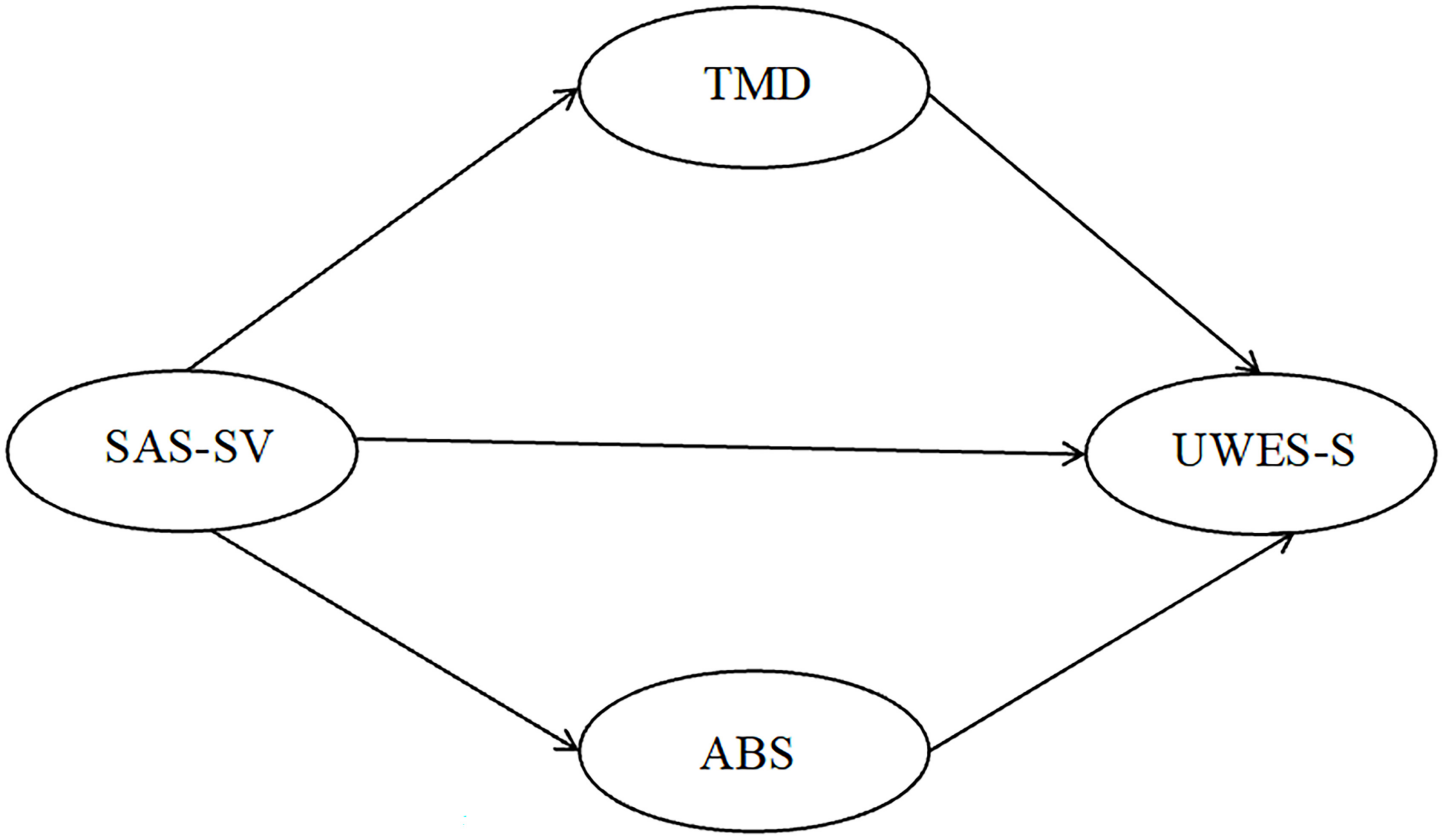
Hypothesized model. SAS-SV, smartphone addiction scale-short version; TMD, time management disposition scale; ABS, Chinese Academic Burnout Scale; UWES-S, Utrecht Work Engagement Scale-Student.

Therefore, to address these gaps, we recruited recruited nursing undergraduates from five universities across three regions in China – including a municipality, an autonomous region, and a province – covering Central, Southwestern, and Northwestern areas. We also constructed a parallel mediation model to examine how smartphone addiction is associated with learning engagement through time management disposition and academic burnout. We hypothesized that: (a) smartphone addiction is negatively associated with learning engagement; (b) time management disposition mediates the relationship between smartphone addiction and learning engagement; and (c) academic burnout mediates the relationship between smartphone addiction and learning engagement (Figure 1). We used a parallel rather than a serial mediation model. This decision was theory-driven: existing literature provides limited justification for a sequential pathway in which smartphone addiction first impairs time management, which in turn exacerbates burnout, and finally relates to reduced learning engagement. It seems more plausible that smartphone addiction may simultaneously relate to disrupted time planning and depleted emotional resources, each independently associated with lower learning engagement. Accordingly, a parallel mediation model was deemed more appropriate for testing our hypotheses.

The present study aims to offer two main contributions. Theoretically, by integrating the time structure perspective and Conservation of Resources theory into a single parallel mediation model, we extend the application of these frameworks to the context of nursing undergraduate education, enriching the understanding of how concurrent pathways operate in the association between smartphone addiction and learning engagement. Practically, by comparing the magnitude of the two indirect effects, our findings may help identify which pathway should be prioritized in intervention design, i.e., whether greater emphasis should be placed on time–management training or burnout alleviation. This may provide empirical evidence for universities to develop targeted intervention strategies to improve the quality of nursing professional training.

## Methods

### Participants

In this multi-center cross-sectional study conducted from May to June 2026, we employed a convenience sampling method to recruit undergraduate nursing students from a university in Hubei Province, a university in Ningxia Hui Autonomous Region, and three universities in Chongqing City. We included: ➀full-time undergraduate student majoring in nursing; ➁voluntary participants who provided informed consent; ➂students with competent communication skills and the ability to independently complete the questionnaires. We excluded: ➀students who were on leave, dropped out, or were unable to participate due to personal reasons; ➁individuals diagnosed with severe physical diseases (e.g., malignant tumors, severe cardiovascular and cerebrovascular diseases) or severe mental disorders (e.g., schizophrenia, major depressive disorder); ➂those currently participating in intervention programs or similar research related to smartphone use, learning behaviors, and mental health.

Sample size estimation was conducted in accordance with conventional methodological criteria, whereby the recommended sample size is generally 5 to 10 times the item count of the longest scale (Liu et al., 2026; Thomas, 2023). In this study, the Time Management Disposition Scale, consisting of 44 items, contained the largest number of questions. Considering a 20% non-response rate, the minimum and maximum required sample sizes were calculated as follows: 44 × (5–10)/80%, yielding a target sample range of 275–550 participants.

### Research instruments

#### General sociodemographic questionnaire

Following a comprehensive literature review, the research team developed a general sociodemographic questionnaire. This questionnaire consisted of 11 indicators: gender, age, grade, only-child status, student leader experience, interpersonal relationship, parental rearing patterns, whether nursing was the first-choice major, satisfaction with teaching quality of the university or college, passion for nursing knowledge after admission, and willingness to engage in nursing after graduation.

#### Utrecht work engagement scale-student (UWES-S)

The UWES-S was used to assess students’ learning engagement levels. This scale was originally formulated by Schaufeli and colleagues (Schaufeli et al., 2002a), and its revised Chinese edition was adapted by Fang et al. (2008). As a validated tool for assessing students’ learning engagement, it covers three subscales: vigor (6 items), dedication (5 items), and absorption (6 items), containing 17 items in total. A 7-point Likert scoring system was adopted, with options varying from 1 (never) to 7 (always). The overall total score ranged from 17 to 119, where higher scores represented better learning engagement. The Chinese version has been extensively applied in domestic nursing student populations with satisfactory psychometric properties (Peng et al., 2025; Wu et al., 2025). In the present study, the Cronbach’s α coefficient of the scale was 0.984.

#### Smartphone addiction scale-short version (SAS-SV)

The SAS-SV comprises 10 items, which are scored on a 6-point Likert scale (Kwon et al., 2013). The total score varies from 10 to 60 points, and higher scores indicate a greater tendency toward problematic smartphone use. This scale was employed to assess the severity of smartphone addiction among participants. Its cut-off values in this study were adopted from the original scale development guidelines, with cut-off scores of ≥31 for male students and ≥33 for female students (Kwon et al., 2013). Previous cross-cultural investigations have verified the suitability and stable measurement characteristics of the Chinese version among domestic college students (Zhao et al., 2022). The Cronbach’s α for this study was 0.967, indicating satisfactory internal consistency reliability.

#### Time management disposition scale (TMD)

The TMD was adapted to the cultural and educational background of Chinese populations (Huang and Zhang, 2001). The scale includes 44 items divided into time value (10 items), time control (24 items), and time efficacy (10 items) sense. All items are rated on a 5-point Likert scale from 1 (strongly inconsistent) to 5 (strongly consistent). The total score ranges from 44 to 220, and higher scores represent better overall time management ability. Regarding interpretation, an average item scores >3 indicates moderate or higher time management levels, while an average score <3 suggests relatively insufficient time management. The TMD has been widely applied among undergraduate nursing students in China with stable psychometric characteristics (Li et al., 2025; Lin et al., 2025). In this study, the Cronbach’s α was 0.959, showing acceptable internal consistency.

#### Chinese academic burnout scale (ABS)

Academic burnout was assessed using the Chinese ABS, which was revised and localized by Lian and his research team (Lian et al., 2005). The scale is composed of 20 items, covering 3 core latent dimensions, including emotional exhaustion (8 items), inappropriate learning behavior (6 items), and reduced professional efficacy (6 items). Among these items, 12 are scored in the positive direction, and 8 adopt reverse scoring settings. All responses are rated on a 5-point Likert scale, with response options ranging from 1 (never) to 5 (always). Higher total scores correspond to more severe academic burnout symptoms. The theoretical critical value of the scale is 60 points. Participants with a total score ≥60 were diagnosed with academic burnout. The ABS has been repeatedly verified to have good psychometric quality and is suitable for investigating nursing students in domestic educational settings (Hu et al., 2024; Liu et al., 2026). In the present study, the Cronbach’s α coefficient of the ABS was 0.921, demonstrating ideal internal consistency reliability.

### Ethics approval

This research was reviewed and approved by the institutional ethics committee (Approval No. CZLL2025-251-001). All procedures were performed in accordance with the ethical guidelines outlined in the Declaration of Helsinki. Prior to completing the online questionnaire, all participants fully understood the research purpose and confirmed voluntary participation by providing an electronic informed consent.

### Data collection

Prior to participant recruitment, the research team communicated the research objectives, implementation procedures, data confidentiality protocols, and voluntary participation principles to relevant college administrative staff at five universities across three regions in China: Chongqing Municipality (Universities A, B, and C), Hubei Province (University D), and Ningxia Hui Autonomous Region (University E). Following administrative authorization, the electronic questionnaire was distributed to eligible nursing undergraduates through official class WeChat groups. The research team developed the online questionnaire using Questionnaire Star, a professional digital survey platform. All participants were informed that participation was voluntary, and refusal or withdrawal would not affect their academic standing. Before accessing the questionnaire, each participant was required to provide electronic informed consent. The anonymous function was activated to ensure privacy and anonymity. The questionnaire took approximately 17 to 22 min to complete.

**FIGURE 2:**
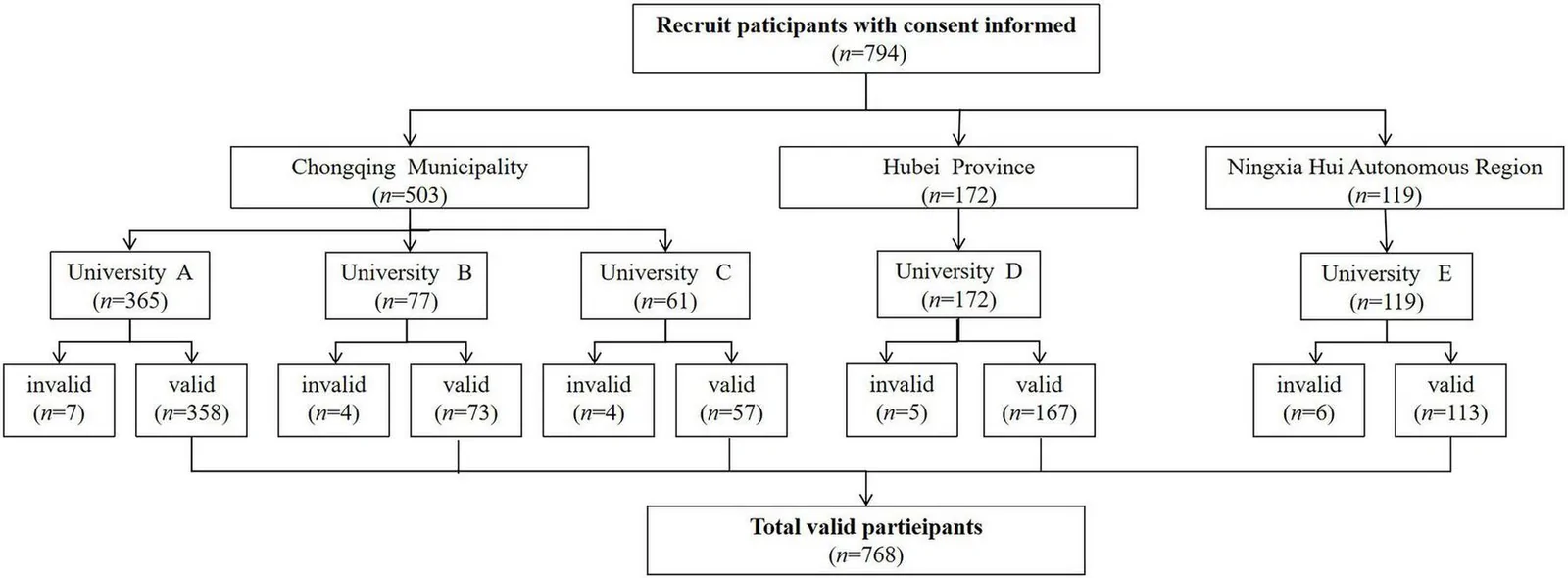
Participant recruitment flow chart.

To ensure data quality, all survey items were set as required responses to avoid missing data. Meanwhile, the IP address limitation function was activated to guarantee that each participant could only submit the questionnaire once, effectively preventing repeated submissions and ensuring data independence. After the completion of data collection, two uniformly trained researchers independently screened all retrieved questionnaires according to unified inclusion criteria. Questionnaires with unreasonable response durations, regular repetitive answering patterns, or obvious logical contradictions were excluded. Overall, 794 questionnaires were distributed across the five participating universities. After excluding invalid responses, 768 questionnaires were retained, yielding an overall response rate of 96.7%. The detailed participant flow for each institution is presented in Figure 2.

### Statistical methods

Statistical analyses were performed using IBM SPSS 25.0 and AMOS 23.0 software. First, descriptive statistics including mean and standard deviations (SD) were calculated to analyze the scores of learning engagement, smartphone addiction, time management disposition and academic burnout. Second, Harman’s single-factor test and single-factor confirmatory factor analysis (CFA) were conducted to examine common method bias (CMB) inherent to self-reported questionnaire data. Third, Pearson correlation analysis was adopted to explore the relationships between research indicators. Finally, we used AMOS 23.0 to establish a structural equation model for parallel mediating effect analysis. The significance level for all statistical tests was set at α = 0.05. Following the mediating effect testing framework (Wen and Ye, 2014), the structural model was estimated via the maximum likelihood method. Several mainstream fit indices were adopted to evaluate model fitness, including *x*^2^/*df*, comparative fit index (CFI), goodness-of-fit index (GFI), Tucker-Lewis index (TLI), incremental fit index (IFI), and root mean square error of approximation (RMSEA). For large-sample research, a *x*^2^/*df* value < 5 is considered acceptable (Lefcheck, 2016). The criteria for adequate model fit were defined as CFI > 0.90, GFI > 0.90, TLI > 0.90, IFI > 0.90, and RMSEA < 0.08. The bootstrap approach was applied to assess indirect effects. Specifically, a mediating effect was statistically significant when the 95% CI of the standardized path coefficient did not contain zero.

## Results

### Common method bias test

Since all data were collected via self-reported questionnaires, CMB could potentially inflate the observed relationships. To minimize this risk, procedural and statistical remedies were implemented following the recommendations of Podsakoff et al. (2012). Procedurally, respondents were assured of anonymity and confidentiality and reverse-coded items were included to reduce response tendencies. Statistically, we first conducted Harman’s single-factor test, which revealed that the first unrotated factor explained 26.9% of the total variance, < 40% (the recommended threshold), suggesting no severe CMB (Podsakoff et al., 2003). However, given the recognized limitations of the Harman test, we further employed a more rigorous single-factor CFA comparison approach. Specifically, the theoretical four-factor measurement model (where each item loads only on its intended construct) was compared against a single-factor model (where all items were forced to load on one common factor). The four-factor model exhibited excellent fit: x^2^/df = 2.864, CFI = 0.984, TLI = 0.974, RMSEA = 0.071. In contrast, the single-factor model produced significantly worse fit: x^2^/df = 7.336, CFI = 0.495, TLI = 0.477, RMSEA = 0.146 (Table 1). The Δ CFI between the two models was 0.489, which exceeded the recommended threshold of 0.10 (Podsakoff et al., 2012; Zhang Q. et al., 2026), indicating that a single common factor cannot account for the covariance structure of the data. These findings suggest that CMB does not pose a serious threat to the validity of our conclusions.

**TABLE 1:** Comparison of model fit for common method bias test (*N* = 768).

Model	*x*^2^/*df*	RMSEA	CFI	RFI	TLI
Four-factor model (theoretical)	2.864	0.071	0.984	0.984	0.974
Single-factor model	7.336	0.146	0.495	0.495	0.477
Recommended threshold	<3	<0.08	>0.90	>0.90	>0.90
Difference	4.472	0.075	0.489	0.489	0.497

The ΔCFI of 0.489 >0.10 indicates that CMB is unlikely to be a substantive concern.

### General characteristics of participants

We included 768 undergraduate nursing students aged 18–25 years, with a mean age of 20.22 ± 1.21 years. Most participants were female (83.6%), non-only children (77.9%), and junior students (38.3%). Overall, 62.0% selected nursing as their first admission major, and 81.8% reported harmonious interpersonal relationships. The companion-oriented parental rearing style was the most common (48.2%), and most students had positive evaluations of college teaching quality. After enrollment, most students felt passionate about nursing expertise, and 71.7% were willing to pursue a nursing career after graduation (Table 2).

**TABLE 2:** Demographic characteristics (*N* = 768).

Variables	Classification	*n* (%)/M ± SD
Age (years)	18-25	20.22 ± 1.21
Gender	Male	126 (16.4)
	Female	642 (83.6)
Only child	No	598 (77.9)
	Yes	170 (22.1)
Grade	Freshman year	205 (26.7)
	Sophomore year	166 (21.6)
	Junior year	294 (38.3)
	Senior year	103 (13.4)
First admission major choice	No	292 (38.0)
	Yes	476 (62.0)
Interpersonal relationship status	Harmonious	628 (81.8)
	Disharmonious	4 (0.5)
	Neutral	136 (17.7)
Parental rearing style	Permissive	226 (29.4)
	Authoritarian	172 (22.4)
	Companion-oriented	370 (48.2)
Satisfaction with teaching quality of the university/college	Very satisfied	304 (39.6)
	Satisfied	358 (46.6)
	Neutral	101 (13.2)
	Dissatisfied	5 (0.6)
Degree of passion for nursing expertise after enrollment	Very passionate	222 (28.9)
	Passionate	281 (36.6)
	Neutral	236 (30.7)
	Unpassionate	29 (3.8)
Intention to engage in nursing career after graduation	Extremely willing	237 (30.9)
	Willing	313 (40.8)
	Neutral	189 (24.5)
	Unwilling	29 (3.8)

M, mean; SD, standard deviation.

### Mean and correlation between major variables

The mean scores of the SAS-SV, TMD, ABS, and UWES-S were 34.10 ± 11.27, 160.80 ± 22.32, 54.26 ± 12.42 and 78.49 ± 19.04, respectively. Descriptive statistics and Pearson correlation coefficients of all variables are presented in Table 3. Pearson correlation analysis indicated that SAS-SV was significantly negatively correlated with TMD (*r* = −0.456, *P* < 0.01) and UWES-S (*r* = −0.551, *P* < 0.01), and significantly positively correlated with ABS (*r* = 0.572, *P* < 0.01). TMD showed a significant negative correlation with ABS (*r* = −0.569, *P* < 0.01) and a significant positive correlation with UWES-S (*r* = 0.692, *P* < 0.01). ABS was significantly negatively correlated with UWES-S (*r* = −0.603, *P* < 0.01).

**TABLE 3:** Description of variables and correlation analysis results (*N* = 768).

Variables	Range	Mean	SD	SAS-SV	TMD	ABS	UWES-S
SAS-SV	10-60	34.10	11.27	1	1	1	1
TMD	64-211	160.80	22.32	−0.456**			
ABS	20-92	54.26	12.42	0.572**	−0.569**		
UWES-S	17-119	78.49	19.04	−0.551**	0.692**	−0.603**	

**P < 0.01. SD, standard deviation; SAS-SV, Smartphone Addiction Scale-Short Version; TMD, Time management disposition scale; ABS, Chinese Academic Burnout Scale; UWES-S, Utrecht Work Engagement Scale-Student.

### Parallel mediating effect

**FIGURE 3:**
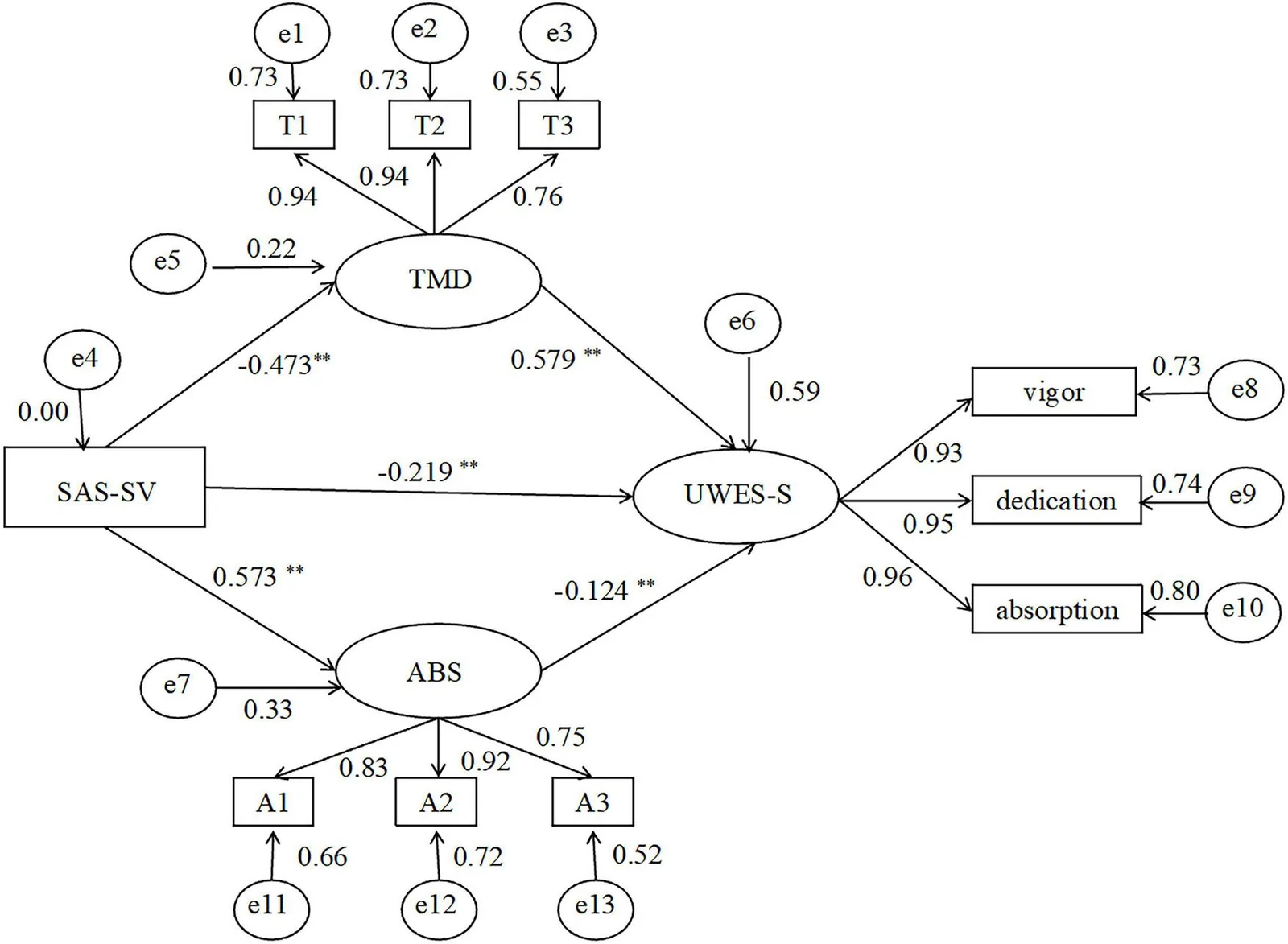
Parallel mediating model of time management disposition and academic burnout. **P < 0.01; SAS-SV, Smartphone Addiction Scale-Short Version; TMD, Time management disposition scale; ABS, Chinese Academic Burnout Scale; UWES-S, Utrecht Work Engagement Scale-Student. T1, time value sense; T2, time control sense; T3, time efficacy sense. A1, emotional exhaustion; A2, inappropriate learning behavior; A3, reduced professional efficacy.

As presented in Figure 3, the measurement model exhibited satisfactory goodness-of-fit with the following indices: x^2^/df = 2.864, RMSEA = 0.071, IFI = 0.984, CFI = 0.984, GFI = 0.967 and TLI = 0.974. All fitness indicators met the recommended standard thresholds. As summarized in Table 4, smartphone addiction showed a significant total association with learning engagement, with a coefficient of −0.564 and a 95% CI ranging from −0.382 to −0.270. Specifically, the direct effect between smartphone addiction and learning engagement was statistically significant at −0.219 (95% CI: −0.347 to −0.108). Additionally, the pooled parallel indirect effect of smartphone addiction on learning engagement was calculated as −0.345, with the 95% CI of −0.254 to −0.138. To determine whether the magnitudes of these two indirect effects differed significantly, we performed a bootstrap test comparing the two pathways. The difference between the two indirect effects was −0.203 (95% CI: −0.167 to −0.064, *P* < 0.001), indicating that the two mediating pathways were significantly different in effect size. This suggests that the pathway through time management disposition played a more dominant mediating role.

**TABLE 4:** Bootstrap test of the mediation effects between smartphone addiction and learning engagement (*N* = 768).

Path	Effect size	SE	*95% CI*	*P*	Percent
Direct: SAS-SV → UWES-S	−0.219	0.019	−0.347 ∼−0.108	<0.001	38.83%
Indirect 1: SAS-SV → TMD → UWES-S	−0.274	0.022	−0.203 ∼−0.118	<0.001	48.58%
Indirect 2: SAS-SV → ABS → UWES-S	−0.071	0.018	−0.078 ∼−0.006	0.019	12.59%
Diff (Indirect 1 vs. Indirect 2)	−0.203	0.027	−0.167 ∼−0.064	<0.001	–
Total indirect effect of SAS-SV on UWES-S	−0.345	0.029	−0.254 ∼−0.138	<0.001	61.17%
Total effect of SAS-SV on UWES-S	−0.564	0.028	−0.382 ∼−0.270	<0.001	100%

SE, standard error; CI, confidence interval; SAS-SV, Smartphone Addiction Scale-Short Version; TMD, time management disposition scale; ABS, Chinese Academic Burnout Scale; UWES-S, Utrecht Work Engagement Scale-Student.

## Discussion

### Influence of smartphone addiction on learning engagement

The structural equation modeling results suggest that smartphone addiction is significantly and negatively associated with learning engagement in nursing undergraduates, indicating that nursing students with higher levels of smartphone addiction tend to show lower levels of learning engagement. This result is consistent with previous studies conducted among college students. In a cross-sectional study of 780 Chinese college students (Peng, 2023), Peng reported that smartphone use was negatively correlated with learning engagement, and students with more severe smartphone addiction exhibited significantly lower levels of learning engagement. Similarly, Li et al. (2019) identified a significant negative association between social networking site addiction and academic engagement among Chinese university students, supporting the view that excessive digital device use reduces learning motivation and classroom participation.

This finding aligns with the limited resource theory proposed by Baumeister and Tice (2007), which states that individuals have finite cognitive resources; when these resources are largely consumed by smartphone use, they become insufficient for learning activities. Zhuang et al. (2023) further noted that learning engagement itself is a key psychological resource for academic success, so excessive smartphone addiction depletes such resources and reduces learning involvement. Additionally, excessive smartphone use often disrupts sleep quality and induces mental fatigue (Deng et al., 2026; Nikolic et al., 2023), which indirectly diminish students’ learning energy and enthusiasm. These findings carry important implications for nursing educators and university administrators.

### Mediating effect of time management disposition

Notably, the results revealed that smartphone addiction was associated with lower learning engagement via its negative correlation with students’ time management disposition. This partial mediating effect of time management disposition explained 48.58% of the total variance, thus confirming our research hypothesis. Consistent with previous findings, smartphone addiction was significantly negatively correlated with time management. Specifically, addictive behaviors may impair college students’ time management, which is regarded as a metacognitive strategy. Multiple studies support this conclusion. As reported in a questionnaire-based study of Chinese undergraduates in Liaoning, Liu et al. (2022) found that students who were better at managing their time scored lower on the internet addiction scale compared with those who struggled with time management. Similarly, a recent cross-sectional study of Chinese students found a significant negative association between mobile phone dependency and time management (Zhang et al., 2025). People who immerse themselves in smartphones may gradually impair their time cognitive system, which may relate to poor time planning and low self-efficacy in managing study schedules.

Our findings suggest that nursing undergraduates’ time management disposition is closely related to their learning engagement, which further supports the self-regulated learning theory (Panadero, 2017). Students with poor time management lack effective self-regulation: they struggle to prioritize academic tasks, underestimate the time required for learning activities, and frequently procrastinate (Khan et al., 2019; Song et al., 2025; Song and Feng, 2017), all of which erode their behavioral, emotional, and cognitive engagement in learning. Conversely, students with strong time management skills are better able to allocate study time reasonably, maintain consistent learning routines, and sustain focus on academic goals, thereby enhancing their overall learning engagement.

### Mediating effect of academic burnout

Notably, academic burnout also acted as a significant partial mediator between smartphone addiction and learning engagement among nursing undergraduates. The mediating effect of academic burnout was statistically significant, accounting for 12.59% of the total variance. Consistent with the Job Demands-Resources (JD-R) framework (Demerouti et al., 2001), smartphone addiction can be conceptualized as a chronic academic “demand” that continuously taxes students’ psychological and attentional resources. Excessive smartphone use consumes limited attentional capacity, increases cognitive load, and disrupts structured study routines, thereby accelerating resource depletion and increasing vulnerability to academic burnout (Chen et al., 2023; Li et al., 2024). Supporting this view, *a prior* study (Li et al., 2026) among Chinese university students has confirmed that smartphone addiction is significantly associated with higher risks of moderate to severe academic burnout, showing a dose-response pattern in which greater addiction correlates with more pronounced burnout symptoms.

In turn, academic burnout is negatively associated with learning engagement. Consistent with the findings of Jeon et al. (2022), lower levels of academic burnout were associated with greater learning engagement in nursing undergraduates. Academic burnout can be viewed as a multidimensional construct encompassing three core components: emotional exhaustion, cynicism, and academic inefficacy (Maslach et al., 2001). Emotional exhaustion refers to a state of extreme mental and emotional fatigue caused by prolonged resource depletion. Cynicism reflects a negative, detached, or indifferent attitude toward learning and related academic activities. Academic inefficacy denotes students’ subjective feelings of incompetence, lack of achievement, and reduced confidence in their academic abilities (Schaufeli and Taris, 2005). Therefore, when students suffer from academic burnout, emotional exhaustion drains their energy and willingness to engage in learning; cynicism weakens their identification with and value of academic tasks; and academic inefficacy further lowers their motivation, persistence, and active participation. Together, these three dimensions reduce students’ emotional investment, cognitive involvement, and behavioral engagement in studying. Findings from previous studies among non-nursing students also support the view that improving academic engagement helps alleviate academic burnout (Qin Z. et al., 2025; Zhang et al., 2023). Taken together, smartphone addiction is associated with elevated academic burnout, which is in turn linked to lower learning engagement among nursing students.

### Comparison of the two mediating pathways

A noteworthy finding of this study is the substantial difference in effect size between the two mediating pathways. The mediating effect of time management disposition was significantly stronger than that of academic burnout. This asymmetry may be attributed to the unique characteristics of nursing education. Nursing students face stricter academic and practical requirements than other disciplines, along with heavy workloads and demanding clinical placements, all of which require strong time management skills (Zhao et al., 2025). When smartphone addiction impairs time management capacity, the resulting disorganization directly undermines students’ ability to meet these demanding requirements, which is associated with lower learning engagement. Furthermore, the disruption of time management by smartphone use tends to be immediate and pervasive, affecting daily study routines, task prioritization, and procrastination behaviors (Wei et al., 2026). In contrast, academic burnout typically develops gradually through prolonged resource depletion (Schaufeli and Taris, 2005), representing a more distal mechanism that may take longer to exert its influence.

### Implications and recommendations

Based on these findings, several practical considerations may be offered for nursing education and student management. First, given the negative association between smartphone addiction and learning engagement, universities and educators may consider developing targeted health education programs to raise students’ awareness of appropriate smartphone use. Life and career education tailored to nursing students’ characteristics might be considered for integration into the curriculum. Interventions to foster students’ sense of meaning in life can also be adopted, such as group counseling (Yang et al., 2023), reflective group activities or case discussions (Thistlethwaite et al., 2012). These measures may help students develop a stronger sense of purpose, which in turn lowers their risk of smartphone addiction triggered by aimlessness.

Second, our findings suggest that time management training and academic burnout support could be promising approaches to protecting students’ learning engagement. Nursing faculty may consider embedding time management strategies into daily teaching or offering dedicated workshops (Lin et al., 2025), and structured training based on the SMART goal-setting principle could be piloted to help students allocate time more efficiently and reduce energy depletion (Li et al., 2025). Similarly, given the mediating role of academic burnout identified in our model, student support programs may benefit from incorporating evidence-based strategies such as progressive muscle relaxation, autogenic therapy, or cognitive behavioral techniques, which have shown short-term positive effects in previous systematic reviews (Gómez-Urquiza et al., 2023; Tating et al., 2023).

Finally, as the mediation of both academic burnout is more distal compared to time management disposition, interventions prioritizing time management training may yield more improvement in learning engagement than those focusing primarily on burnout alleviation. However, this does not diminish the importance of addressing academic burnout, which remains a significant and independent mediating mechanism. These recommendations are derived from our correlational findings and existing literature, and their direct applicability to nursing students should be confirmed through future intervention studies.

### Limitations and prospects

This study has some limitations that should be acknowledged. First, as a cross-sectional study, it cannot establish causal relationships between smartphone addiction, time management disposition, academic burnout, and learning engagement. Future longitudinal studies could further examine potential chain or interactive effects between the two mediating pathways. Second, the use of convenience sampling may have introduced selection bias, as participants were recruited from five universities based on accessibility rather than through random selection. Although we attempted to enhance sample diversity by recruiting from three geographically distinct regions across China, the sample may not be fully representative of all nursing undergraduates. Therefore, the generalizability of our findings should be interpreted with caution. Third, CMB cannot be completely ruled out, as all data were collected via self-reported questionnaires at a single time point. Although we implemented procedural remedies and statistical tests to minimize this risk, the possibility of residual bias remains. Future studies could incorporate objective measures such as actual smartphone usage tracking or academic performance records. Last but not least, variables such as mental health, sleep quality, and socioeconomic status influence both smartphone addiction and learning engagement in previous studies (Qin X. et al., 2025; Xu et al., 2025; Zhang Q. et al., 2026), but were not included in our analysis. Future research could incorporate these factors to more precisely assess the role of smartphone addiction in learning engagement.

## Conclusion

This study constructed a parallel mediation model and revealed that smartphone addiction is not only directly associated with lower learning engagement, but also exhibits indirect correlational links via poorer time management disposition and elevated academic burnout. This finding not only quantifies the intensity of the relationships among variables but also provides a novel empirical perspective for understanding nursing education issues in the digital era.

## Data Availability

The raw data supporting the conclusions of this article will be made available by the authors, without undue reservation.
